# Mammalian Ste-20-like Kinase 1/2 (MST1/2) Inhibitor XMU-MP-1: A Potential Compound to Improve Spermatogenesis in Mouse Model of Diabetes Mellitus

**DOI:** 10.3390/biomedicines12112513

**Published:** 2024-11-03

**Authors:** Bella Amanda, Zakiyatul Faizah, Cennikon Pakpahan, M. Aminudin Aziz, Berliana Hamidah, Faisal Yusuf Ashari, Delvac Oceandy

**Affiliations:** 1 Department of Biomedical Sciences, Faculty of Medicine, Universitas Airlangga, Surabaya 60132, Indonesia; zakiyatul-f@fk.unair.ac.id (Z.F.); cennikon.pakpahan@fk.unair.ac.id (C.P.); azizandro@gmail.com (M.A.A.); berliana-hamidah@fk.unair.ac.id (B.H.); faisalyusufashari@fk.unair.ac.id (F.Y.A.); 2 Airlangga University Teaching Hospital, Universitas Airlangga, Surabaya 60115, Indonesia; 3 Division of Cardiovascular Sciences, Faculty of Biology, Medicine and Health, University of Manchester, Manchester M13 9PT, UK; delvac.oceandy@manchester.ac.uk

**Keywords:** spermatogenesis, diabetes mellitus, male infertility, MST1, Hippo pathway, good health and well-being

## Abstract

**Background/Objectives:** Spermatogenesis is a key process in male reproduction that, if it does not happen correctly, can lead to infertility, with diabetes being one of the most prevalent causes of spermatogenesis disruption. Currently, there is a lack of research examining the potential benefits of targeting cell proliferation to enhance spermatogenesis in this condition. XMU-MP1 has been identified as an inhibitor of MST1, a core component of the Hippo pathway, which is anticipated to promote proliferation and regeneration. This study aims to evaluate the effects of XMU-MP1 treatment on sperm and testicular characteristics in mice. **Methods:** We used the STZ-induced diabetic mouse model to investigate the impact of administering XMU-MP1 on testicular tissue and sperm parameters. This study compared the seminiferous tubules, specifically focusing on the diameter of the seminiferous tubule, the thickness of the seminiferous tubule epithelium, the ratio of the thickness of the seminiferous tubule epithelium to the diameter of the seminiferous tubules, and the lumen diameter of the seminiferous tubules. We also conducted a comparison of sperm parameters, including sperm concentration, progressive motility, total motility, total motility, and morphology. **Results:** XMU-MP1-treated mice had a larger spermatogenesis area and better sperm motility than control mice. Diabetic mice treated with XMU-MP1 also showed a trend toward improvements in the spermatogenesis area, sperm concentration, sperm motility, and sperm morphology, although these improvements were not statistically significant. **Conclusions:** XMU-MP1 serves as a potential compound to improve spermatogenesis in mice.

## 1. Introduction

Approximately 15% of couples worldwide are affected by infertility, with men being responsible for 50% of infertility cases [1]. Infertility is the condition characterized by the failure to conceive a pregnancy after engaging in regular sexual intercourse for a period of one year without the use of any form of contraception. A more significant issue is the extensively documented global decline in sperm counts observed in recent decades [2]. Interruption of spermatogenesis is one factor that may contribute to a decline in sperm count. In the presence of high blood sugar levels, endothelial cells take in more glucose, leading to an overproduction of reactive oxygen species (ROS) in the mitochondria. This excessive ROS production causes oxidative damage and triggers the activation of the inflammatory signaling pathway in endothelial cells. As a result, a significant amount of inflammatory cytokines is released into the bloodstream [3].

Spermatogenesis is a key process that plays a fundamental role that, if it does not happen correctly, can lead to infertility. The process includes cell specification, cell migration, mitotic and meiotic cell division, differentiation, and ultimately maturation. Only when these steps occur in a precise order, within a specified environment, and without any mistakes, will a sufficient number of fully developed haploid spermatozoa be generated. This is necessary for the fertilization of an egg and subsequent embryonic development [4].

Streptozotocin (STZ)-induced diabetic mice’s testes and epididymal sperm are subjected to significant oxidative stress during the early diabetic phase, which may appear to contribute to the development of testicular dysfunction, resulting in altered steroidogenesis and impaired spermatogenesis [5]. A study conducted on mice with diabetes found that there were decreases in the amounts of testosterone, follicle-stimulating hormone (FSH), and luteinizing hormone (LH) in their blood [6]. Hyperglycemia also causes significant disruptions in lipid metabolism, specifically the breakdown of triglycerides, fatty acyl esters of cholesterol, and steroid hormones. Research has also demonstrated that maintaining a healthy lipid metabolism is crucial for optimal spermatogenesis [7]. Hyperglycemia triggers oxidative stress (OS) and causes apoptosis, along with other harmful effects [8]. An increase in the expression of the pro-apoptotic proteins Bax, Bad, and c-Jun N-terminal kinases was observed in rats with STZ-induced hyperglycemia. This rise was found to be associated with an elevation in germ cell death [9,10].

At the moment, the treatment options for male infertility, particularly in cases of hypogonadism, are mostly focused on hormone therapy to stimulate spermatogenesis. The final recourse for males suffering from a diminished sperm count or azoospermia, especially those who are unable to undergo hormone therapy, is to undergo surgical sperm retrieval for later utilization in assisted reproductive technology. Currently, there is a lack of research examining the potential benefits of targeting cell proliferation to enhance spermatogenesis.

The Hippo pathway is a series of signals that control a diverse range of biological activities, including cell proliferation and differentiation, regulation of organ size, and the ability to regenerate. YAP/TAZ serves as an essential downstream mediator of the Hippo pathway, capable of relocating to the nucleus to stimulate TEAD-mediated gene expression associated with cellular growth and proliferation [11]. The Hippo pathway has been identified in regulating cell proliferation in multiple organs, including the heart, liver, lungs, and pancreas [11]. The Hippo pathway has been targeted to improve liver regeneration [12], heart remodeling [13], and provide beneficial effects in mouse models of diabetes [14,15]. Studies indicate that the Hippo signaling pathway plays several roles in the testis, such as contributing to testicular development [16], germ cell differentiation [17], and Sertoli cell proliferation [18]. The Hippo signaling pathway may also influence LH release and Sertoli cell proliferation in response to FSH stimulation [19,20]. However, there is still no study that has reported whether targeting a Hippo pathway component can improve spermatogenesis or sperm quality, particularly after injury to the testicles resulting from diabetes mellitus. XMU-MP1 is a selective and potent inhibitor of MST1, a component of the Hippo signaling pathway [21]. XMU-MP1 has been reported to enhance the proliferation of hepatocytes and small intestine epithelial cells while reducing apoptosis, fibrosis, and pathological hypertrophy in the heart [13,21]. MST1 is expressed in pancreatic beta cells and plays a role to the pathophysiology of diabetes mellitus [22]. In this study, we investigated whether treatment with the MST1 inhibitor, XMU-MP1, would improve spermatogenesis and the sperm phenotype in a mouse model of diabetes mellitus.

## 2. Materials and Methods

### 2.1. In Vivo Experiments

Experimental animal research was carried out according to ethical principles in animal research. The research procedure was approved by the Ethics Committee of the Faculty of Medicine, Universitas Airlangga.

We used 12-week-old Balb/c mice obtained from a certified laboratory animal breeder supplier, Pusat Veteriner Farma (Pusvetma), Surabaya. The average body weight (BW) of the mice was 19.86 g at the start of the experiments. Mice were kept in the experimental animal facility within the Faculty of Medicine (Universitas Airlangga) under standard housing conditions for laboratory animals. The laboratory animals’ temperatures were controlled at 19–22 °C, with a humidity of 40–65% and a 12 h light/dark cycle. Mice were fed a standard chow diet.

Mice were injected with STZ (BioWorld, Ohio, USA) at a dose of 20 mg/kg BW/day, for 5 consecutive days, to create the diabetes mellitus model. Additionally, diabetic model mice received unlimited d10 solution. Random blood glucose was measured at basal levels before STZ injection (day 0), and at days 10, 17, 24, and 32 following the first STZ injection. The criterion of diabetes was a random blood glucose (RBG) level more than 200 mg/dL at day 17 after the first STZ injection. Mice were then divided into 4 groups: (i) control group; (ii) XMU-MP1 (ChemScene, New Jersey, USA) group (mice treated with XMU-MP1 only); (iii) diabetic group (mice treated with STZ); and (iv) XMU-MP1-treated diabetic group (diabetic mice treated with XMU-MP-1). Mice in group (iv) were injected with vehicle (DMSO) with the same volume as XMU-MP-1. DMSO (Merck, Darmstadt, Germany) was used a standard solvent for XMU-MP1. Therefore, control mice were treated with DMSO only to match with the treated group. XMU-MP-1 at a dose of 1 mg/kg BW/day and an equal volume of vehicle (DMSO) were given intraperitoneally starting at day 10 after the first day of STZ injection for a total of 21 days. Mice were sacrificed at day 32 after blood glucose measurement.

We conducted the analysis in three steps: the 1st part of the study looked at the effect of XMU in control (normal) mice, especially the sperm and testicular phenotypes in response to XMU-MP1 treatment. The second part looked at the effect of STZ-induced hyperglycemia on sperm and testicular phenotypes. The third part looked at the effect of XMU-MP1 treatment on STZ-induced diabetic mice on sperm and testicular phenotypes.

### 2.2. Sperm Analysis

Sperm were obtained from the epididymis. Each epididymis was carefully cut to remove adipose and other tissue. Sperm were removed from the epididymis by injecting 1 mL of PBS fluid into the epididymis orifice and then left for approximately 1 min. The suspension was mixed gently before carrying out the sperm analysis. Sperm motility was assessed under a 400× microscope by counting 200 sperm in two separate preparations, for a total of 400 sperm per mouse. Progressive motility is defined as the ability of sperm to move fast forward, whereas sperm displaying movement limited to a single location are categorized as having non-progressive motility. Total motility is the sum of progressive and non-progressive motility. Sperm concentration was determined using the improved Neubauer chamber in accordance with the 5th WHO semen analysis guidelines. The evaluation of sperm morphology was conducted on a minimum of 200 sperm cells. Normal morphology is established when the sperm exhibit a typical head, midpiece, and tail [23].

### 2.3. Histological Analysis of Testis Tissues

Testis tissues collected at the end of experiment were fixed using 4% normal buffered formalin for 24 h. Tissues were processed overnight using a Krisme automated tissue processor and were then embedded in paraffin wax. The histological sections were prepared at 5 μM thickness using a rotary microtome (Leica 2125, Chicago, IL, USA). Analysis of the seminiferous tubule area were conducted using an Olympus BX-41 microscope with 400× magnification. Images were analyzed using the ImageJ software (v1.52, NIH, Bethesda, MD, USA).

### 2.4. Data Analysis

This study compared the seminiferous tubules, specifically focusing on the diameter of the seminiferous tubule, the thickness of the seminiferous tubule epithelium, the ratio of the thickness of the seminiferous tubule epithelium to the diameter of the seminiferous tubules, and the lumen diameter of the seminiferous tubules. We also conducted a comparison of sperm parameters, including sperm concentration, progressive motility, total motility, total motility, and morphology. Data are presented as the mean ± standard deviation (SD). Statistical analysis was performed using GraphPad Prism software v. 9.5.0 (GraphPad Software, San Diego, CA, USA). The unpaired *t*-test was used to compare significance among groups; a *p*-value < 0.05 was considered to be significant.

## 3. Results

### 3.1. Treatment with XMU-MP1 Improves Sperm Motility

**Figure 1:**
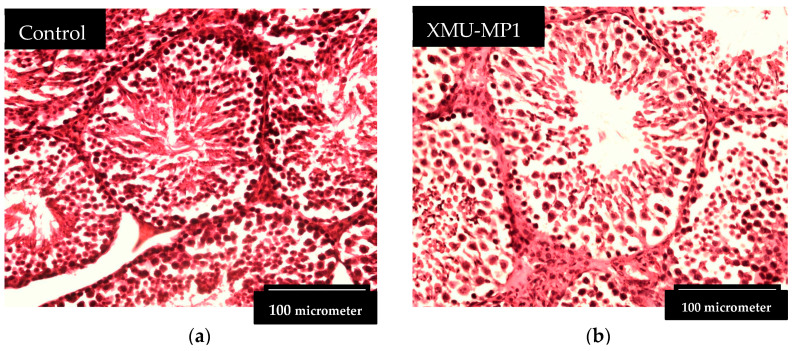
(**a**) A representative image of the control group’s testis histopathology; (**b**) A representative image of the XMU-MP1-treated group’s testis histopathology. The diameter of the seminiferous tubules in the XMU-MP1-treated mice group is bigger than that in the control group.

**Figure 2:**
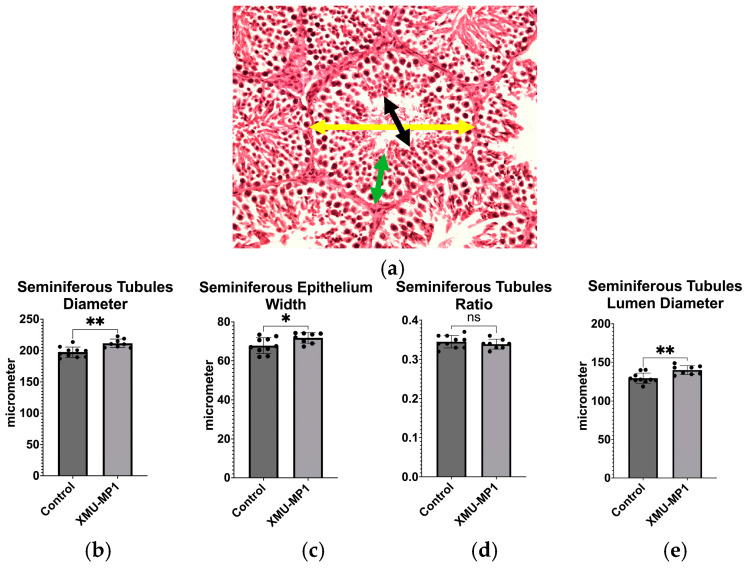
(**a**) An image depicting a cross-section of a seminiferous tubule stained with hematoxylin and eosin. The yellow arrow indicates the diameter of the seminiferous tubule, the green arrow indicates the seminiferous tubule epithelium width, and the black arrow represents the diameter of the seminiferous tubule lumen; (**b**) Comparison of seminiferous tubule diameter between the control group and the XMU-MP1-treated group; (**c**) Comparison of seminiferous tubule epithelium width between the control group and the XMU-MP1-treated group; (**d**) Comparison of seminiferous tubule ratio between the control group and the XMU-MP1-treated group; (**e**) Comparison of seminiferous tubule lumen diameter between the control group and the XMU-MP1-treated group. (control, *n* = 10; XMU-MP1, *n* = 8). * *p* < 0.05, ** *p* < 0.01, ns = not significant.

The Hippo pathway plays a crucial role in the control of cell proliferation. To understand if inhibition of the Hippo pathway would increase spermatogenesis, we performed experiments by injecting mice with XMU-MP1 as an inhibitor of MST-1. Following treatment with XMU-MP1, we discovered a significantly larger seminiferous tubule diameter (*p* = 0.0012), seminiferous epithelium width (*p* = 0.0304), and seminiferous tubule lumen diameter (*p* = 0.0030) in the XMU-MP1-treated mice compared to controls (Figure 1 and Figure 2).

**Figure 3:**
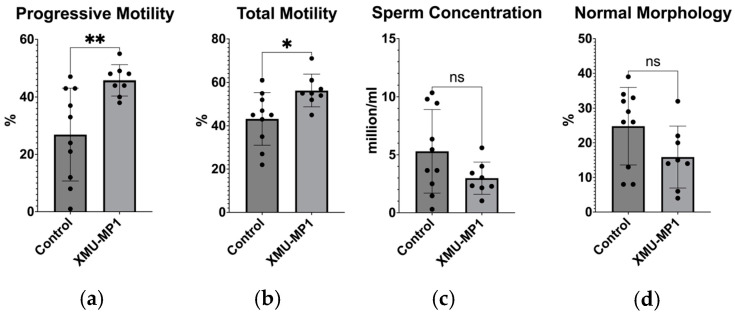
(**a**) Comparison of sperm cells’ progressive motility between the control group and the XMU-MP1-treated group; (**b**) Comparison of sperm cells’ total motility between the control group and the XMU-MP1-treated group; (**c**) Comparison of sperm concentration between the control group and the XMU-MP1-treated group; (**d**) Comparison of sperm cell’s morphology between the control group and the XMU-MP1-treated group. (control, *n* = 10; XMU-MP1, *n* = 8). * *p* < 0.05, ** *p* < 0.01, ns = not significant.

We examined sperm motility to determine the capacity of the sperm to reach the egg. After giving mice XMU-MP1 therapy for 21 days, we examined the mice’s sperm motility. Mice were sacrificed and the sperm were isolated from the epididymis. After the sperm were isolated, we analyzed the sperm parameters according to the WHO manual for semen analysis. Sperm motility was divided into three categories: progressive motility, which refers to the sperm’s ability to travel swiftly and straight; non-progressive motility, which refers to the sperm moving in the same spot; and immotile motility, which refers to the sperm’s complete lack of movement. We observed better sperm motility in the XMU-MP1 treatment group compared to controls. We found an 18.85% improvement in progressive motility (*p* = 0.0063) and 13.05% increase in total motility (*p* = 0.0175) (Figure 3). However, we did not find any changes in sperm concentration and morphological parameters. Overall, our data suggested that XMU-MP1 enhanced sperm motility.

### 3.2. Effects of STZ-Induced Hyperglycemia on Testicular Phenotypes and Spermatogenesis

**Figure 4:**
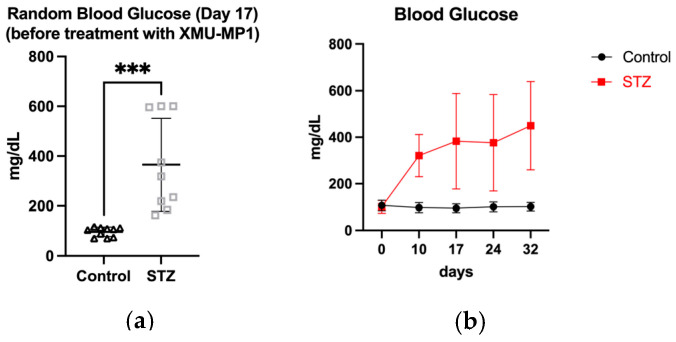
(**a**) Analysis of random blood glucose (RBG) at day 17 after STZ injection (before XMU-MP1 treatment). (control, *n* = 10; STZ, *n* = 9). *** *p* < 0.001 (**b**) Random blood glucose measurement in control and diabetes mellitus groups.

Diabetes mellitus is often associated with decreased sperm parameters and increased incidence of infertility. To investigate the effects of diabetes mellitus on spermatogenesis, we analyzed the testicular phenotypes in the mouse model of diabetes mellitus. Random blood glucose was measured at day 0 before STZ injection and days 10, 17, 24, and 32 following the first STZ injection. From the start of the observation to the end, the random blood glucose levels were steady in the control group, while they increased in the diabetes mellitus group, exceeding 200 mg/dL (Figure 4).

**Figure 5:**
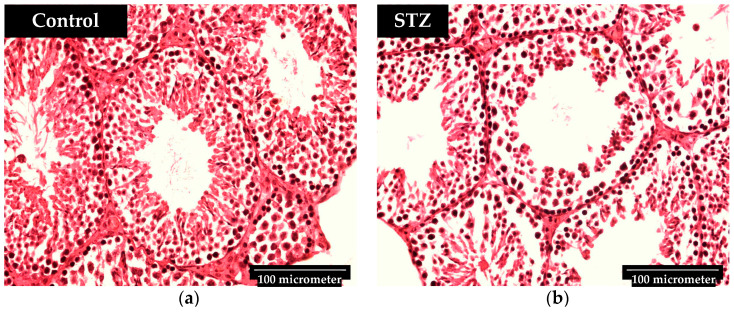
(**a**) A representative image of the control group’s testis histopathology; (**b**) A representative image of the STZ-treated group’s testis histopathology.

**Figure 6:**
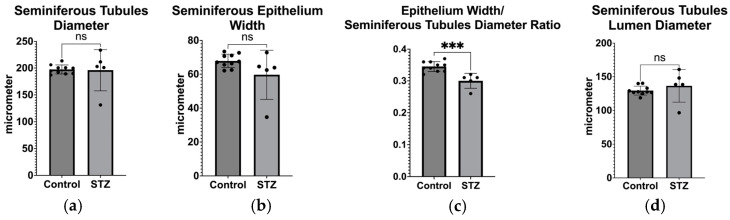
(**a**) Comparison of seminiferous tubule diameter between the control group and the STZ-treated group; (**b**) Comparison of seminiferous tubule epithelium width between the control group and the STZ-treated group; (**c**) Comparison of seminiferous tubule ratio between the control group and the STZ-treated group; (**d**) Comparison of seminiferous tubule lumen diameter between the control group and the STZ-treated group. (control, *n* = 10; STZ, *n* = 5). *** *p* < 0.001, ns = not significant.

**Figure 7:**
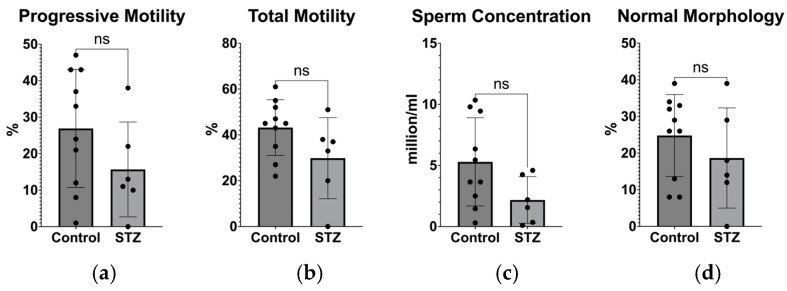
(**a**) Comparison of sperm cells’ progressive motility, (**b**) total motility, (**c**) sperm concentration, and (**d**) sperm morphology between the control group and the STZ-treated group. (control, *n* = 10; STZ, *n* = 5). ns = not significant.

As shown in Figure 5, the diameter and epithelium width of the seminiferous tubules in the control and diabetes mellitus groups did not differ significantly, as indicated by the analysis of the testicular histological sections. We then looked at the ratio of the seminiferous tubule epithelium’s width to its diameter, which revealed a significant reduction of the spermatogenesis region in the STZ-treated group compared to the controls. Other seminiferous tubules parameters were not different between the two groups analyzed (Figure 6). From the sperm phenotype analysis, we did not observe statistically significant differences, but we found a trend of reduced sperm concentration, progressive motility, total motility, and morphology (Figure 7).

### 3.3. XMU-MP1 May Have a Positive Impact on Sperm Parameters of STZ-Induced Diabetic Mice

**Figure 8:**
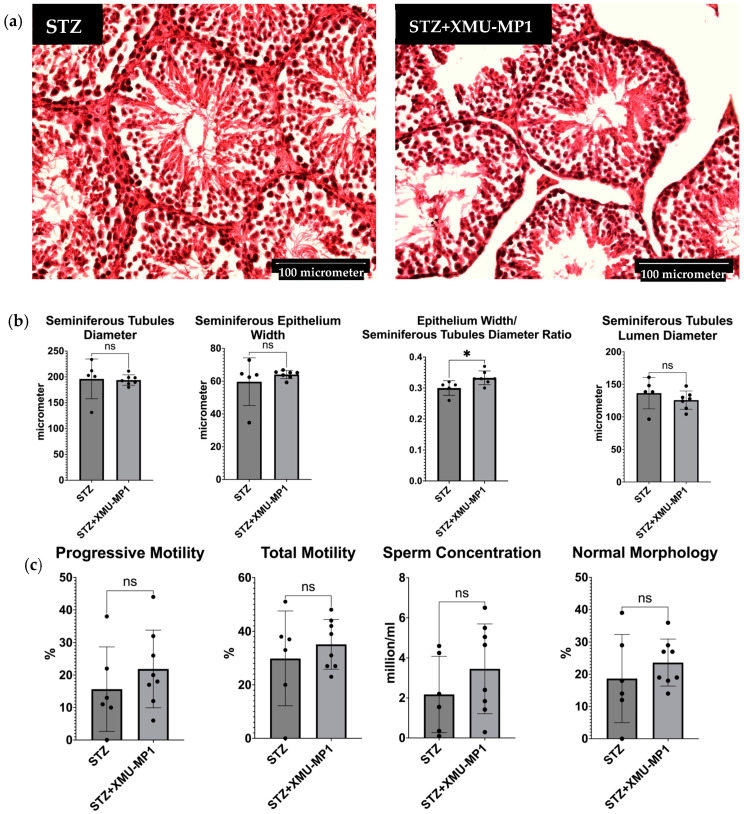
(**a**) Representative images of the STZ-treated group’s and STZ+XMU-MP1-treated group’s testis histopathologies. The diameter of the seminiferous tubules in the STZ+XMU-MP1-treated group was smaller than in the STZ-treated group. However, the ratio of the epithelium width of the seminiferous tubule to its diameter was greater in the STZ+XMU-MP1-treated group; (**b**) Comparison of seminiferous tubule diameter, seminiferous epithelium width, seminiferous tubule ratio, and seminiferous tubule lumen diameter between the STZ-treated group and STZ+XMU-MP1-treated group (STZ, *n* = 5; STZ+XMU-MP1, *n* = 7); (**c**) Comparison of sperm parameters between the STZ-treated group and STZ+XMU-MP1-treated group (STZ, *n* = 5; STZ+XMU-MP1, *n* = 8). * *p* < 0.05, ns = not significant.

Since treatment with Hippo pathway inhibitor XMU-MP1 increased the spermatogenesis area and improved sperm motility, we hypothesised that treatment of diabetic mice with XMU-MP1 would improve the testicular and sperm phenotypes. To address this question, we treated diabetic mice with XMU-MP1. Analysis of histological sections demonstrated a trend of denser structure and a better ratio of the seminiferous tubule epithelium width to the diameter when compared to the diabetes mellitus group receiving no treatment, with *p* = 0.0329 (Figure 8). It is interesting to note that sperm parameters, including motility, concentration, and shape, exhibited an upward trend in the DM group receiving XMU-MP1 therapy, despite the fact that the difference was not statistically significant. This demonstrates that XMU-MP1 may have a positive impact on sperm characteristics in STZ-induced diabetic mice.

## 4. Discussion

In this study, it was found that XMU-MP1 may increase spermatogenesis and sperm motility in wild-type mice. Although there was a trend of improvement, XMU-MP1 did not significantly improve the sperm phenotype and spermatogenesis area in the diabetic mouse model. Diabetes might adversely affect male fertility through many routes. Diabetes might impact the endocrine regulation of spermatogenesis and sperm metabolism by decreasing the synthesis of follicle-stimulating hormone (FSH) and luteinizing hormone (LH) [24,25]. Decreased levels of leptin, which plays a metabolic role in the brain, can also create disruptions in the hypothalamic–pituitary–testicular axis [25,26]. Diabetes can alter the structure of the seminiferous tubules by reducing their diameter and thinning the germinal epithelium [27], which aligns with the outcomes seen in this investigation. Animal investigations have demonstrated that there is a disturbance in the characteristics of sperm, such as their concentration, motility, and shape [28]. Although not statistically significant, our study also found a declining trend in sperm concentration, motility, and morphology in diabetic mice. Nevertheless, other research indicated that diabetes does not have any impact on the concentration and shape of sperm in patients [29]. Diabetes directly impacts the sperm ultrastructure as well [30]. Increased oxidative stress in the testicles is one of the causes of a decrease in sperm parameters in diabetics [31,32]; as a result, regulating the patient’s diabetes and giving antioxidants are now the primary methods of treating male infertility. New ways for treating infertility related to diabetes are required to boost the success of pregnancy programs by increasing sperm parameters.

The Hippo pathway is known as a chief regulator of cell proliferation and regeneration. The Hippo pathway in mammals comprises several essential components, including MST1/2, protein Salvador homologue 1 (SAV1), MOB1A/B, large tumor suppressor kinase ½ (LATS1/2), Yes-associated protein 1 (YAP), WW-domain-containing transcription regulator 1 (TAZ), and the transcriptional enhanced associated domain (TEAD) family [11]. MST1/2 are serine/threonine kinases that promote the interaction of MOB1A/B with LATS1/2. MST1/2 form heterodimers with SAV1, which is necessary for MST1/2 to phosphorylate SAV1, MOB1, and LATS1/2 kinase. LATS1/2 inhibit the nuclear localization of YAP and TAZ by directly phosphorylating them, restricting the transcriptional action of its coactivating component. The activation of MST1/2 protein serves as a starting signal for the Hippo pathway [33]. Many studies have been conducted on many tissues and organs that highlight the importance of the Hippo route, such as the pancreas, lungs, muscles, liver, breast, and heart [34,35,36,37,38,39]. Pharmacological targets of the Hippo pathway have been developed, for example, XMU-MP1, which has been successful in several areas, with the hope of creating possibilities to discover treatments that specifically target the Hippo pathway for various disorders. XMU-MP1, when combined with insulin treatment, alleviated myocardial dysfunction [40] through modulation of the Mst1/AMPK pathway, and reperfusion treatment with XMU-MP1 also reduced myocardial I/R injury and restored myocardial function in mice [41]. XMU-MP1 may have a protective effect on cartilage deterioration, as demonstrated in mice research [42]. Furthermore, XMU-MP1 can enhance glucose tolerance in diabetic mice [14].

In this study, we tried to analyze the effect of XMU-MP1 treatment in WT mice. The data were consistent with the current understanding that XMU-MP1-treated mice have a larger spermatogenesis area and improved sperm parameters. However, when we treated the diabetic mice, we did not observe significant improvements, although there were partial improvements in the spermatogenesis area, sperm concentration, sperm motility, and sperm morphology. The obtained results could be because the diabetes model was still mild. Aside from that, the XMU-MP1 therapy was only given for 21 days, not the full 35-day mouse spermatogenesis cycle. There may also be drug interactions between STZ and XMU-MP1, resulting in suboptimal treatment. Another potential cause is the altered vascularization in STZ-treated mice, which may impede the delivery of treatment to the targeted region, leading to insufficient therapeutic efficacy. So far, the Hippo pathway has been shown to be pro-mitotic [43], but it may have no effect on meiosis.

Several limitations in this study include the diabetes period being too short. Therefore, it is possible that the intervention may not have resulted in substantial impairment to testes in mice with diabetes. In addition, the brief duration of XMU-MP1 therapy, which was not completed for a full cycle of sperm production, may have impacted the outcomes.

## Data Availability

Data are contained within the article.
